# A Circulating MicroRNA Signature as a Biomarker for Prostate Cancer in a High Risk Group

**DOI:** 10.3390/jcm4071369

**Published:** 2015-07-07

**Authors:** Brian D. Kelly, Nicola Miller, Karl J. Sweeney, Garrett C. Durkan, Eamon Rogers, Killian Walsh, Michael J. Kerin

**Affiliations:** 1 Department of Surgery, Clinical Science Institute, National University of Ireland, Galway, Ireland; E-Mails: nicola.miller@nuigalway.ie (N.M.); karljsweeney@gmail.com (K.J.S.); michael.kerin@nuigalway.ie (M.J.K.); 2 Department of Urology, Galway University Hospital, Galway, Ireland; E-Mails: Garrett.Durkan@mailn.hse.ie (G.C.D.); emacruairi@me.com (E.R.); kilian.walsh@gmail.com (K.W.)

**Keywords:** prostate cancer, circulation, microRNA

## Abstract

Introduction: Mi(cro)RNAs are small non-coding RNAs whose differential expression in tissue has been implicated in the development and progression of many malignancies, including prostate cancer. The discovery of miRNAs in the blood of patients with a variety of malignancies makes them an ideal, novel biomarker for prostate cancer diagnosis. The aim of this study was to identify a unique expression profile of circulating miRNAs in patients with prostate cancer attending a rapid access prostate assessment clinic. Methods: To conduct this study blood and tissue samples were collected from 102 patients (75 with biopsy confirmed cancer and 27 benign samples) following ethical approval and informed consent. These patients were attending a prostate assessment clinic. Samples were reverse-transcribed using stem-loop primers and expression levels of each of 12 candidate miRNAs were determined using real-time quantitative polymerase chain reaction. miRNA expression levels were then correlated with clinicopathological data and subsequently analysed using qBasePlus software and Minitab. Results: Circulating miRNAs were detected and quantified in all subjects. The analysis of miRNA mean expression levels revealed that four miRNAs were significantly dysregulated, including *let-7a* (*p* = 0.005) which has known tumour suppressor characteristics, along with *miR-141* (*p* = 0.01) which has oncogenic characteristics. In 20 patients undergoing a radical retropubic-prostatectomy, the expression levels of *miR-141* returned to normal at day 10 post-operatively. A panel of four miRNAs could be used in combination to detect prostate cancer with an area under the curve (AUC) of 0.783 and a PPV of 80%. Conclusion: These findings identify a unique expression profile of miRNA detectable in the blood of prostate cancer patients. This confirms their use as a novel, diagnostic biomarker for prostate cancer.

## 1. Introduction

Prostate cancer is the most commonly diagnosed non-cutaneous malignancy in men and is the second leading cause of cancer death [1]. It is estimated that up to one in six men will be diagnosed with prostate cancer during their lifetime [2]. Clinicians use a combination of a digital rectal examination (DRE) and a prostate specific antigen (PSA) and a transrectal ultrasound guided prostate biopsy (TRUS) to detect prostate cancer. However, prostate cancer screening trials, such as The Prostate, Lung, Colorectal and Ovarian cancer screening trial (PLCO) and the European Randomised Study of Screening for Prostate cancer (ERSPC) trials, have highlighted that despite an increase in the diagnosis of prostate cancer using these tests, there is still no clear improvement in mortality [3,4]. In addition, PSA, a frequently used biomarker for the detection of prostate cancer, is limited by its lack of sensitivity and specificity for prostate cancer and therefore not considered an ideal biomarker. As a result, a search for a novel, minimally invasive, clinically relevant biomarkers for the detection of prostate cancer is required.

mi(cro)RNAs are small non-coding endogenous RNA molecules that vary in length from 18–25 nucleotides. There are numerous dysregulated miRNAs that are implicated in the pathogenesis of cancer and have been shown to regulate gene expression and function at the transcriptional and post-transcriptional level. They play a pivotal role in the expression of up to 60% of human genes [5]. miRNAs can be up or down-regulated, with up-regulation of oncogenic miRNAs and down-regulation of tumour suppressor miRNAs are demonstrated in a variety of malignancies. Dysregulation of miRNA has been associated with the pathogenesis of different cancers and approximately up to 50% of miRNA genes are located in cancer-related genomic regions [6]. Despite their small size miRNAs are extremely stable molecules and have been identified and quantified in RNA extracted from formalin fixed paraffin embedded tissue samples that have been stored for many years [7]. miRNAs are remarkably stable in the circulation and are protected from endogenous ribonuclease (RNase) activity and from variations in pH and temperature [8].

A number of studies have identified that there are numerous miRNAs that are dysregulated in prostate cancer tissue [9,10,11,12]. More recently, studies have identified that dysregulated miRNAs are also detectable in the circulation of patients with differing malignancies [13,14]. Specific to prostate cancer, Mitchell *et al.* identified that epithelial cancers release miRNAs into the circulation and that *miR-141* could identify those patients with metastatic prostate cancer from healthy controls [8]. As a result, miRNAs have the potential to be a novel, stable, non-invasive biomarker.

The primary aim of this study was to investigate if a miRNA signature was detectable that was unique to patients with prostate cancer in comparison with patients with benign prostatic histology attending a prostate assessment clinic. Secondary aims were to assess if there is a correlation between circulating levels of miRNAs and increasing risk stratification of prostate cancer as per the D’Amico risk stratification and also if the miRNA signature returned to normal after a radical prostatectomy [15].

## 2. Materials and Methods

### 2.1. Patients

Ethical approval was granted for the collection of blood samples and tissue samples by the Ethics committee at Galway University Hospital. Patients were recruited from the rapid access prostate assessment clinic (RAPAC) at Galway University Hospital tertiary referral cancer centre. Informed written consent was obtained from each patient prior to the collection of samples. Men were referred to the RAPAC if they had an elevated PSA, an abnormal DRE or a family history of prostate cancer. Histological diagnosis was made following a 12 core TRUS biopsy of the prostate.

### 2.2. Blood Collection and Storage

Whole blood samples were prospectively obtained from patients prior to TRUS biopsy and collected in 10 mL Ethylenediaminetetraacetic acid (EDTA) tubes. Samples were collected between September 2009 and March 2011 and stored at 4 °C until RNA extraction occurred. Whole blood was selected for analysis as this has previously been shown to have high yields of RNA and higher expression levels of miRNAs [16]. Relevant clinicopathological data was obtained from a prospectively maintained prostate cancer database.

### 2.3. Selected miRNA Targets

miRNAs are ideal molecules for a blood-based biomarkers for the detection of cancer, as they are dysregulated in carcinogenesis and are highly stable in both tissue and in blood samples. Various studies have documented the differential expression of miRNAs in the circulation of patients with cancer when compared with non-cancer patients and healthy controls, making miRNA an ideal non-invasive biomarker. Not all miRNAs that are dysregulated in prostate cancer tissue are released into the circulation. The exact mechanism by which miRNAs are released still remains unclear. miRNAs could be passively leaked or actively secreted into the circulation. Passive leakage can occur by tissue degradation associated with malignancy, through this mechanism miRNAs could be released into the circulation in an energy free mechanism.

A panel of 12 miRNAs were selected for miRNA expression profiling. They were selected on the basis of previously reported dysregulated expression levels in prostate tumour samples and in the circulation of prostate and other cancers and also based on information gleaned from previous studies within the Discipline of Surgery at NUI Galway [8,11,14,17,18,19]. The miRNAs investigated included *miR-16*, *-21*, *-34a*, *-141*, *-143, -145, -155*, *-125b*, *-221*, *-375*, *-425* and *let7a* (see Table 1).

**Table 1:** The 12 miRNAs selected for the expression profiling in the circulation.

Dysregulated miRNA	Source	References
*let-7a*	Blood, Tissue	Heneghan *et al.* [14]; Volinia *et al*. [19]; Porkka *et al*. [11]; Tong *et al*. [31]
*miR-21*	Blood, Tissue	Zhang *et al*. [30]; Yaman Agaoglu *et al*. [26]; Volinia *et al*. [19]; Ozen *et al*. [32]
*miR-34a*	Tissue	Ambs *et al*. [18]; Ozen *et al.* [32]
*miR-125b*	Blood, Tissue	Mitchell *et al*. [8]; Porkka *et al*. [11]; Ozen *et al*. [32]; Tong *et al*. [31]; Schaefer *et al*. [33]; Spahn *et al.* [34]
*miR-141*	Blood, Tissue	Mitchell *et al*. [8]; Brase *et al*. [17]; Porkka *et al*. [11]
*miR-143*	Blood, Tissue	Mitchell *et al*. [8]; Porkka *et al*. [11]; Tong *et al*. [31]
*miR-145*	Blood, Tissue	Heneghan *et al*. [14]; Porkka *et al*. [11]; Ozen *et al*. [32], Ambs *et al*. [18]; Tong *et al*. [31]; Schaefer *et al*. [33]
*miR-155*	Blood	Heneghan *et al*. [14]
*miR-221*	Blood, Tissue	Yaman Agaoglu *et al*. [26]; Zheng *et al*. [35]; Porkka *et al*. [11]; Ambs *et al*. [18]; Ozen *et al*. [32]; Tong *et al*. [31]; Schaefer *et al*. [33]; Spahn *et al*. [34]
*miR-375*	Blood, Tissue	Brase *et al*. [17]; Schaefer *et al*. [33]
*miR-16*	Blood, Tissue	Lawrie *et al*. [21]; Heneghan *et al*. [14]; Huang *et al*. [22]; Liu *et al*. [20]; Wong *et al.* [24]
*miR-425*	Tissue	Chang *et al*. [25]

### 2.4. RNA Extraction

Total RNA was extracted from 102 samples using TRI Reagent BD (Molecular Research Centre Inc., Cincinnati, OH, USA), from 1 mL of whole blood. The concentration of the RNA was ascertained using Nanodrop spectrophotometry (Nanodrop ND-1000 Technologies Inc., Wilmington, DE, USA). Extracted RNA was subsequently stored at −80 °C.

### 2.5. Reverse Transcription and RQ-PCR

100 ng of total RNA was reversed transcribed to cDNA using stem loop primers specific to each target miRNA of interest. RQ-PCR was performed using Taqman primers and probes (Applied Biosystems, Foster City, USA) on a 7900 HT Fast Real-Time PCR System (Applied Biosystems). RQ-PCR was performed on all samples in triplicate and interassay controls were used throughout. The threshold standard deviation for each of the replicates was taken at 0.3 for both samples and interassay controls.

PCR amplification efficiencies were calculated for each individual miRNA using the following equation: E = (10**^−^**^1/slope^ − 1) × 100. The efficiency threshold was calculated at +/−10% across a 10-fold dilution series across five points. The relative miRNA expression levels (∆∆Ct) were calculated relative to endogenous controls, *miR-16* and *miR-425*. These were selected from a panel of miRNAs based on their stability and minimal variation across 66 benign and malignant blood samples (data not shown).

### 2.6. Statistical Analysis

QBasePlus was utilised to calculate the miRNA expression levels. Statistical analysis was performed using Minitab v16. The 2 sample t-test was used to compare the miRNA expression levels of cancer cases with benign cases. An analysis of variance (ANOVA) was used to analyse miRNA expression levels across factors of interest. A *p*-value of <0.05 was considered as significant with a Bonferroni correction. Binary logistic regression analysis was used to calculate an area under the curve (AUC) for combined miRNAs to determine their sensitivity and specificity.

## 3. Results

### 3.1. Patient Demographics

A total of 102 patients were selected at random and included in this study. Following TRUS biopsy 75 men were subsequently diagnosed with prostate cancer (median age 64 years, median PSA 7.4 µg/L) and 27 had a benign histological finding (median age 65 years, median PSA 7.45 µg/L). There was no significant difference between the PSA levels of the benign or cancer group, as most patients were referred with an elevated PSA level (see Table 2). Twenty eight patients had Gleason score 6, 34 had Gleason score 7, six had Gleason score 8 and seven patients had Gleason score 9 prostate cancer. In terms of risk stratification, there were 28 men with low-risk, 11 with intermediate-risk and 36 with high-risk prostate cancer (see Table 3). Within the benign group, men with a persistently elevated PSA underwent a second biopsy. We appreciate that there is a high cancer detection rate within this group and there is a high incidence of high grade disease which is a fair representation of the men referred to our service.

**Table 2:** Patient demographics.

	Benign	Cancer
Numbers (102)	27	75
Age		
Median	65 years	64 years
Range	48–80 years	48–85 years
PSA (prostate specific antigen)		
Median	7.45 µg/L	7.4 µg/L
Range	1–77 µg/L	1.42–52.24 µg/L

**Table 3:** Histology, D’Amico risk stratification and dysregulated miRNAs.

Histology	Numbers (102)	Risk Stratification	Numbers (75)
Benign	27	Low	28
3 + 3	28	Intermediate	11
3 + 4	20		
4 + 3	14	High	36
4 + 4	6		
4 + 5	7		
**miRNA**	**Up or Down Regulated**	***p* Value**	**AUC (area under the curve)**
*let-7a*	↓	0.005	0.678
*miR-141*	↑	0.014	0.655
*miR-145*	↑	0.01	0.634
*miR-155*	↑	0.01	0.624
*miR-375*	↑	0.075	0.651

**Figure 1:**
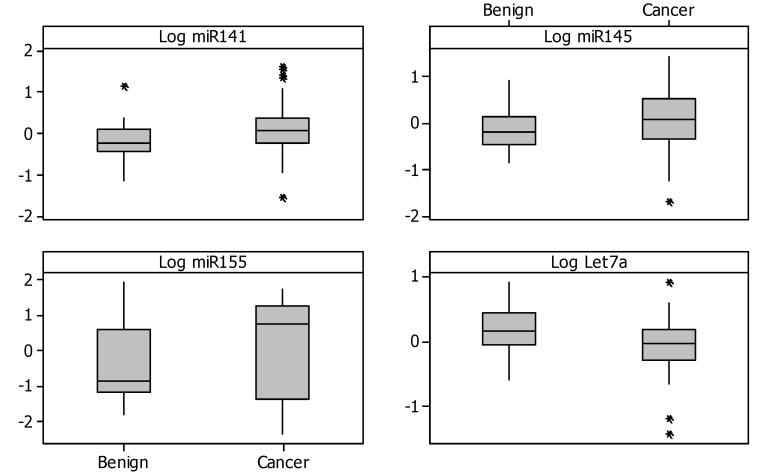
Boxplots of the four dysregulated miRNAs.

Of the 12 miRNAs quantified, there were four significant miRNAs (*p* < 0.05) (see Table 3). Three of these miRNAs were up-regulation of oncomirs (*miR-141*, *-145* and *-155*) and down-regulation of the tumour-suppressor *let7a* in patients with prostate cancer as compared with benign disease (see Figure 1). There was a trend towards significance for the oncomir *miR-375*, which was upregulated in patients with prostate cancer (*p* = 0.07).

### 3.2. miRNAs as a Predictive Marker of Prostate Cancer

Of the 12 miRNAs investigated, four miRNAs were able to distinguish those with prostate cancer (*n* = 75) from those without (*n* = 27). To further investigate the diagnostic potential of these miRNAs, receiver operator curves (ROC) were generated for each and the AUCs were calculated as the measure of their accuracy. These include *let7a, miR-141, -145* and *-155*, with an AUC of 0.678, 0.655, 0.634 and 0.624 respectively (see Table 3). The oncomir *miR-141* had a sensitivity of 94% and a positive predictive value (PPV) of 73%. The tumour-suppressor *let7a* had a sensitivity of 93% and a positive predictive value (PPV) of 70%.

### 3.3. miRNAs in Combination

Using regression analysis, expression patterns were analysed in combination using the miRNAs as a diagnostic panel to improve upon the sensitivity. Using the four miRNAs mentioned above (*let-7a*, *miR-141*, *-145* and *miR-155*) the sensitivity improved to 97%, with a PPV of 80% and an AUC of 0.783.

### 3.4. Risk Stratification

The expression levels of *let7a* decreased from benign to low-risk and to intermediate-risk prostate cancer, as per the D’Amico risk stratification, with a similar mean expression levels for both intermediate-risk and high-risk prostate cancer were observed (*p* = 0.04). There was a significant upregulation of *miR-141* in relation to D’Amico risk stratification (*p* = 0.023). The expression levels of *miR-145* and *miR-155* increased as the risk increased, however, this only approached significance (*p* = 0.1 and *p* = 0.115, respectively).

### 3.5. Post-Operative miRNAs

Twenty men had pre-operative and post-operative blood taken to quantify miRNA levels. The four miRNAs mentioned above were also quantified post-operatively. The blood was collected the day prior to surgery and at mean post-operative day 10 (range day 7–22). The post-operative expression levels of *miR-141* reduced considerably to levels similar to those of the patients with benign histological findings. However, the other three miRNAs did not return to the benign levels.

## 4. Discussion

This study demonstrates that four miRNAs are significantly dysregulated in the circulation of patients with prostate cancer. Combining these miRNAs to identify a unique prostate cancer miRNA signature revealed a biomarker panel with an AUC of 0.783. A study investigating the expression profile of patients with gastric cancer using five circulating miRNAs in combination had a sensitivity of 80%, a specificity of 81% and an AUC of 0.879 [20].

To date there are a limited number of studies examining the expression profile of miRNAs in the circulation of patients with prostate cancer. Examining these papers reveals that there are a variety of different techniques used for RNA extraction and from different blood mediums such as whole blood, serum and plasma. Our institution has recently published on the variability of miRNA levels in whole blood, serum and plasma, and identified that whole blood contains a higher yield of miRNAs by RQ-PCR [16].

*Mir-16* and *miR-425* were found to be stably expressed in the circulation of all patients with little variability. As a result both were selected for use as endogenous controls. *miR-16* has been used as a normaliser in a many studies investigating levels of miRNAs in whole blood, serum, plasma and tissue [16,21,22,23,24]. To our knowledge, this is the first published evidence of *miR-425* being used as an endogenous control in the circulation, but has previously been described as a suitable endogenous control in tissue [25].

The oncomir *miR-141*, when quantified in circulation, Ha the ability to identify those men with prostate cancer with an AUC of 0.655. This highlights a potential clinical use of miRNAs in the identification of patients with malignancy in a group deemed to be clinically high risk due to an elevated PSA. Mitchell *et al.* have previously reported that *miR-141* could differentiate those with prostate cancer with an AUC of 0.9, although all 25 of the prostate cancer patients had metastatic disease [8]. Levels of *miR-141* has been shown to increase as the stage of disease progresses from organ confined disease, to locally advanced disease and on to metastatic prostate cancer [17,26]. However, similar to Mitchell *et al.* and using a RNA extraction technique from serum, Mahn *et al.* encountered difficulties in the detection of *miR-141* in the sera [27]. This further highlights the variability in results from different extraction methods, different blood products and the use of different endogenous controls. There is also evidence to support that RNAse activity is increased in the serum of prostate cancer patients however Mitchell *et al.* identified that circulating miRNAs are stable against RNAse activity [8,28].

In this study *let7a* was found to be significantly downregulated in prostate cancer patients. *Let7a* has the ability to act as both a oncogene and tumour suppressor and is dysregulated in a number of malignancies [14]. This is first reported downregulation of *let-7a* in the circulation of patients with prostate cancer, this also concurs with previous papers citing *let-7a* as downregulated in prostate cancer tumour tissue [11,19].

We have identified that mean expression levels of *miR-141* significantly reduce post-operatively. Previous studies have also highlighted that the expression levels of oncomirs return to normal after oncological surgery, this has been identified in patients undergoing radical prostatectomy, mastectomy and colonic resection [16,27,29]. Zhang *et al.* identified that *miR-21* was upregulated in patients with hormone refractory prostate cancer and *miR-21* levels were reduced in patients who responded to docetaxel chemotherapy as compared to those hormone refractory patients who were resistant to chemotherapy [30]. This highlights the potential use for miRNAs as biomarkers for treatment response and also as prognostic markers.

Using the four miRNAs (*miR-141, -145, -155* and *let7a*) in combination yielded a sensitive biomarker panel with an AUC of 0.783. Given a sensitivity of 97%, with few false negative results, illustrates that quantifying a panel of miRNAs in the circulation has the potential to reduce unnecessary TRUS biopsies from being performed, allows for risk stratification in active surveillance protocols and in the future may help with choices of therapeutic intervention for physicians, surgeons and patients. A limitation of this study is that men within the benign group with a high PSA may indeed have an undiagnosed prostate cancer.

## 5. Conclusions

This study has identified a panel of four miRNAs that have diagnostic potential superior to that of PSA and DRE for the detection of prostate cancer. Three miRNAs were observed to be upregulated and one miRNA downregulated in association with prostate cancer. The expression levels of two of these miRNAs were altered as stage of disease increased. miRNAs, due to their detection, dysregulation and stability in blood, hold immense promise as future, novel, non-invasive biomarkers for prostate cancer.
